# Transcriptional profile of isoproterenol-induced cardiomyopathy and comparison to exercise-induced cardiac hypertrophy and human cardiac failure

**DOI:** 10.1186/1472-6793-9-23

**Published:** 2009-12-09

**Authors:** Cristi L Galindo, Michael A Skinner, Mounir Errami, L Danielle Olson, David A Watson, Jing Li, John F McCormick, Lauren J McIver, Neil M Kumar, Thinh Q Pham, Harold R Garner

**Affiliations:** 1 McDermott Center for Human Growth and Development, University of Texas Southwestern Medical Center, Dallas, Texas, USA; 2 Department of Surgery, University of Texas Southwestern Medical Center and Children's Medical Center of Dallas, Texas, USA

## Abstract

**Background:**

Isoproterenol-induced cardiac hypertrophy in mice has been used in a number of studies to model human cardiac disease. In this study, we compared the transcriptional response of the heart in this model to other animal models of heart failure, as well as to the transcriptional response of human hearts suffering heart failure.

**Results:**

We performed microarray analyses on RNA from mice with isoproterenol-induced cardiac hypertrophy and mice with exercise-induced physiological hypertrophy and identified 865 and 2,534 genes that were significantly altered in pathological and physiological cardiac hypertrophy models, respectively. We compared our results to 18 different microarray data sets (318 individual arrays) representing various other animal models and four human cardiac diseases and identified a canonical set of 64 genes that are generally altered in failing hearts. We also produced a pairwise similarity matrix to illustrate relatedness of animal models with human heart disease and identified ischemia as the human condition that most resembles isoproterenol treatment.

**Conclusion:**

The overall patterns of gene expression are consistent with observed structural and molecular differences between normal and maladaptive cardiac hypertrophy and support a role for the immune system (or immune cell infiltration) in the pathology of stress-induced hypertrophy. Cross-study comparisons such as the results presented here provide targets for further research of cardiac disease that might generally apply to maladaptive cardiac stresses and are also a means of identifying which animal models best recapitulate human disease at the transcriptional level.

## Background

Physiological increase in cardiac muscle mass occurs normally during development, during pregnancy, and in response to sustained exercise [1]. Conversely, pathological cardiac hypertrophy is an adaptive response to increased pressure load, such as with hypertension or in the setting of aortic stenosis, or it may be associated with an inherited disease characterized by thickening of the left ventricle and disarray of myocytes [2]. Moreover, regional hypertrophy can result as a consequence of myocardial infarction in response to ischemic heart failure. Cardiac failure remains a major source of human morbidity and mortality in the United States [3]. The disease has multiple specific etiologies, and much research has been performed to elucidate some of the multiple molecular pathways important in the development of myocardial failure [4,5]. Many animal models of cardiac failure have been devised, and they have played an important role in understanding this complicated disease [6]. However, in many cases the specific method of inducing heart failure in animals does not obviously correlate with common human diseases [7].

For example, some of the animal models of heart failure are induced by pharmacologic agents to induce a direct cardiomyopathy; however, it is relatively rare for the human heart to be injured through such a mechanism [7]. Even the surgical occlusion of a coronary artery to induce acute ischemia of the animal heart does not precisely model the more common chronic ischemia that characterizes the failing human myocardium [8]. Although creation of a pressure overloaded heart by aortic banding might very closely model the pressure overloaded human heart, such as might be seen in aortic valvular disease, it is likely that there are significant differences in myocardial response to acute pressure load induced in the animal when compared to the more chronic human situation. Therefore, it is difficult to know which animal models best exemplify particular types of heart failure in humans.

Genetic expression array analysis allows the characterization of tissue by assessing the expression patterns of thousands of genes. Such investigations of diseased human myocardium have been used to elucidate which molecular pathways are altered in particular etiologies of heart failure [9,10]. In some cases, the differences in genetic expression patterns elucidated by microarray analysis are significant enough for clustering algorithms to distinguish different categories of human heart failure. We hypothesized that the sensitivity of this approach will allow us to determine how closely a common animal model of cardiac failure recapitulates the genetic milieu of the human disease.

We induced cardiac hypertrophy in mice using isoproterenol, which has been shown to induce significant cardiac dysfunction associated with a high mortality rate (up to 80%) [7]. We subjected the myocardium to genetic expression array analysis. Genes demonstrating altered expression compared to normal control myocardium and myocardium that was hypertrophied following an exercise regimen were compared to various other animal models of cardiac hypertrophy (e.g., aortic banding, hormone treatment, and high salt diet) and also human diseased myocardial expression data obtained from publicly available databases. We reasoned that microarray genetic expression analysis of a murine heart failure model might exhibit similarities at the genetic expression level with some category of human disease, and would therefore help determine which animal models correlate most precisely with particular human diseases.

## Results

### Analysis of pathological and physiological cardiac hypertrophy mouse models

**Figure 1:**
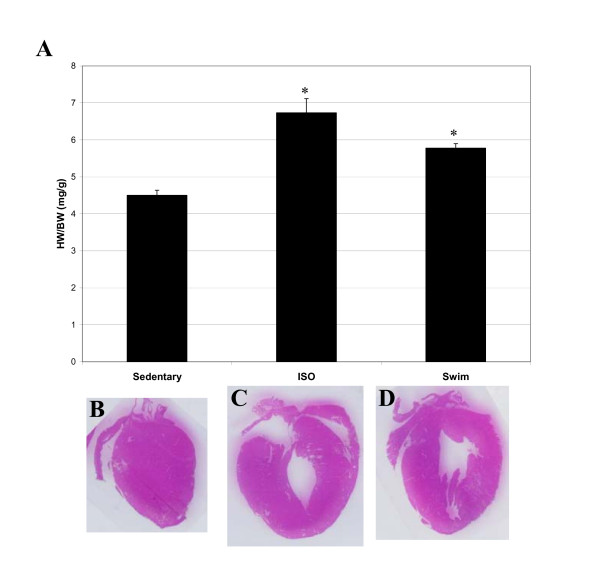
**ISO-treatment and exercise induce enlargement of mouse hearts**. Top - Heart Weight to Body Weight ratios (HW/BW) for sedentary, ISO-treated or exercised mice. Values are represented as mean ± S.E.M of all mice (8-week old C57BL/6J males). There were 6 mice each in the sedentary group, ISO group, and exercised group. The asterisk denotes statistical significance based on Student's t test for ISO-treated or exercised mice compared to sedentary controls (p value < 0.01). Bottom- Representative histological coronal sections of hearts of sedentary control (A), ISO-treated (B), and exercised (C) C57BL/6J mice. As shown, ISO-treated and exercised mice exhibited enlarged heart masses compared to sedentary mice.

**Figure 2:**
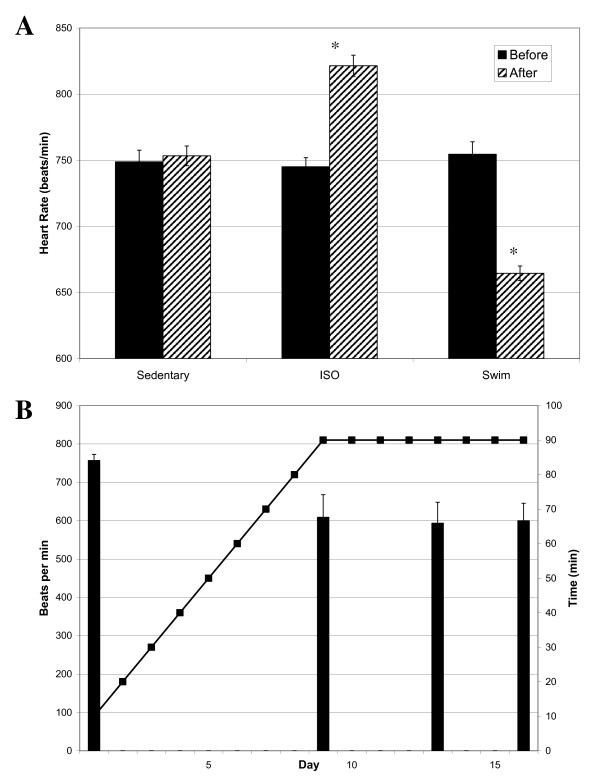
**Heart rate is increased under ISO treatment, but reduced under exercise training**. A) Comparison of heart rates in sedentary, ISO-treated, and exercised mice. Heart rates were measured before the experiment and on the day of sacrifice (for ISO-treated) or throughout the experiment (for exercised mice). Each heart rate is the average of at least three measurements from rested mice (i.e., swimming mice heart rates were taken in the morning before exercise). Asterisk indicates statistical significance when compared to sedentary control mice, based on one way ANOVA (p value = 0.008, n = 4 and p value = 0.007, n = 6 for ISO and swim mice, respectively). B) Heart rates taken at various time intervals are indicated by bars (mean ± S.E.M), with beats per minute given on the left ordinate. Minutes swam (twice per day) with 5 min/day increases are shown as a line of interconnected points, with minutes per training session displayed on the right ordinate.

Isoproteronol (ISO) administration and chronic exercise training successfully created cardiac hypertrophy in experimental animals. As shown in Fig. 1A, heart weight/body weight ratios increased significantly (p values > 0.01, n = 6) for both the ISO-treated (6.7 ± 0.85 mg/g) and exercised mice (5.8 ± 0.40 mg/g), compared to sedentary animals (4.5 ± 0.31 mg/g). However, the increase in heart weight was much more pronounced for mice with ISO-induced cardiac hypertrophy than for exercised mice. Similar results were obtained when heart weight/tibia length ratios were compared (7.1 ± 0.32 mg/mm for sedentary mice versus 10.8 ± 1.1 mg/mm and 7.9 ± 0.41 mg/mm for ISO-treated and exercised mice, respectively, p value < 0.01, n = 6). Hearts were also visibly larger, particularly after ISO-treatment, as determined by examination of H&E stained cryosections (Fig. 1B-D). While both ISO treatment and exercise induced enlargement of the heart, only the pathological hypertrophy model resulted in an increased heart rate (745 ± 15 beats/min before ISO treatment and 821 ± 16 beats/min after ISO-treatment, p value 0.02, n = 4; Fig. 2A). This finding may be related to treatment with the beta-agonist isoproterenol, and may not be unique to myocardial hypertrophy. Exercised mice, on the other hand, experienced a profound reduction in heart rate (754 ± 19 beats/min before exercise and 665 ± 13 after swimming, p value 0.002, n = 6; Fig. 2A) that was evident within the first 10 days of the swimming regimen (Fig. 2B).

**Figure 3:**
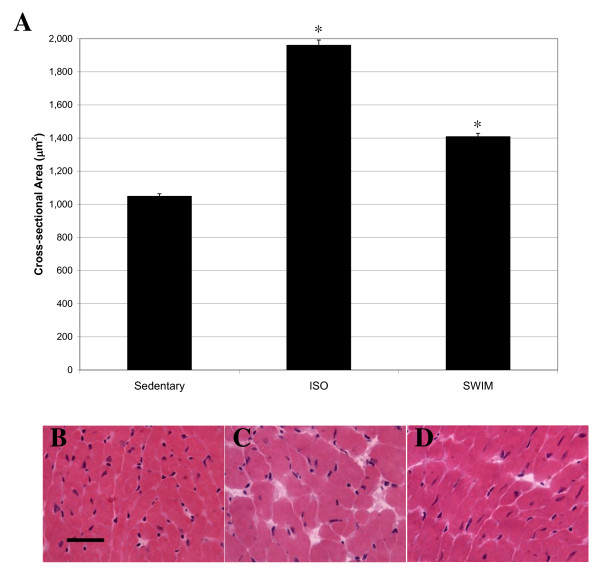
**ISO-treatment and exercise induce enlargement of cardiomyocytes**. A) 2-dimensional cell surface area of stained cardiomyocytes was measured using ImageJ software. At least 5 images were taken of the left-ventricle of three different hearts (for each group). Bars represent mean ± S.E.M of at least 400 cells. Asterisks indicate statistical significance after application of one-way ANOVA (p value < 0.0001). H&E stained sections of the left ventricle of a representative sedentary (B), ISO-treated (C), and exercised (D) mouse are also shown. Black scale bar = 60 μm.

**Figure 4:**
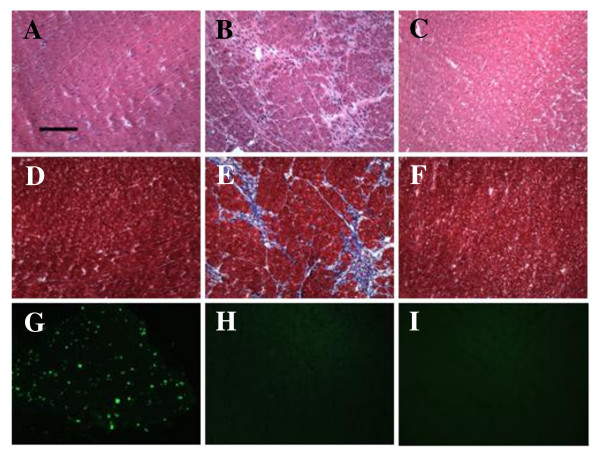
**ISO treatment induces fibrosis but not apoptosis in mouse hearts**. H&E stained sections of the left ventricle of a representative sedentary (A), ISO-treated (B), and exercised (C) mouse are shown. Similar sections were stained with Masson trichrome to assess fibrosis in sedentary (D), ISO-treated (E), and exercised (F) mouse hearts. TUNEL staining indicated that there was no detectable apoptosis in these same hearts. A positive control (mouse thymus) for TUNEL staining is shown in panel G, and representative ISO-treated and exercised mouse hearts (left ventricles) are shown in panels H and I, respectively. TUNEL staining of sedentary mouse hearts was similar to ISO-treated and exercised mice (no apoptosis detected; data not shown). Black scale bar = 120 μm.

To determine whether the increase in heart size was due to increased myocyte size or increased numbers of cardiomyocytes, we quantified cell cross-sectional area in H&E stained heart cryosections. Each cell examined had a circularity (height/width) = 0.90, and > 400 cells were measured for each sample type (i.e., sedentary, exercised, and ISO-treated). As expected, myocyte cross-sectional area was significantly increased in mice treated with ISO, compared to sedentary control mice (Fig. 3A), and cardiomyocytes generally appeared thicker in ISO-treated (Fig. 3C) than in sedentary (Fig. 3B) mouse hearts, when examined individually. Cardiomyocytes from exercised hearts were also larger than those from sedentary mice (Fig. 3A), although the increase in size was less dramatic (Fig. 3D), compared to ISO-treated mice. We also assessed the levels of fibrosis and apoptosis in sedentary, exercised, and ISO-treated hearts (Fig. 4). As shown by H&E and trichrome staining, there was significant fibrosis detected in ISO-treated hearts (Fig. 4B and 4E) that was not observed in sedentary (Fig. 4A and 4D) or exercised (Fig. 4C and 4F) mouse hearts. Conversely, there was no evidence of apoptosis in any of the experimental groups apart from the positive control (mouse thymus), as measured by TUNEL assay (Fig. 4G-I). Considered together, these data indicated that the two models induced the desired effects: maladaptive and beneficial increases in heart mass representing pathological and physiological hypertrophy, respectively.

### Expression profiles of ISO-treated and swim-exercised mice

**Figure 5:**
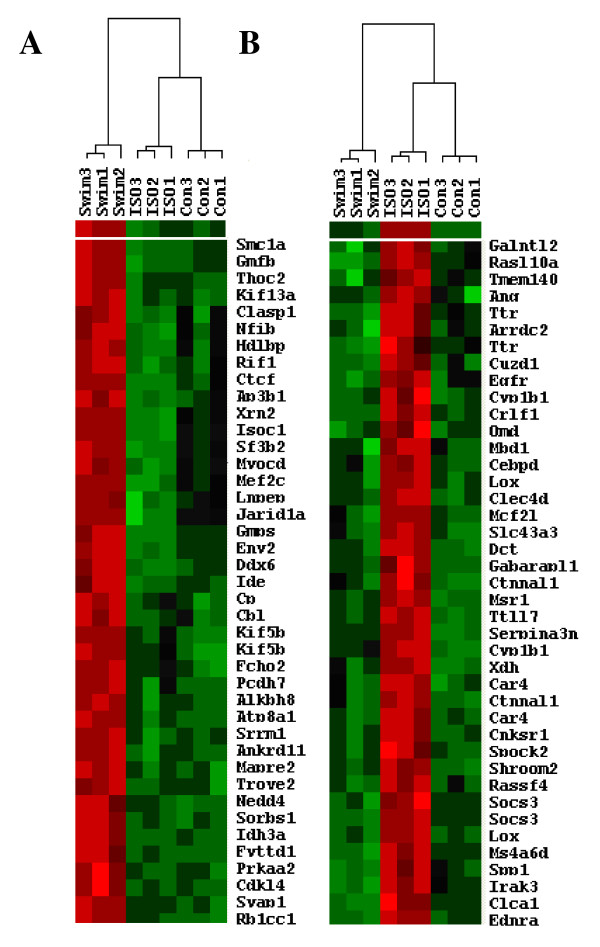
**ISO-treatment an exercise induce differential transcriptional profiles**. Hierarchical clustering of genes altered in mouse hearts in response to exercise (**A**) or ISO treatment (**B**). Bright red indicates the highest normalized signal values, bright green represents the lowest signal values, and black represents median signal values. The heat map was produced by clustering normalized signal values (exported from GeneSifter) using Cluster/Treeview software. Abbreviations used are as follows: Swim = exercised mice, ISO = isoproterenol-treated mice and Con = sedentary control mice. Replicates samples are indicated by number (1 -3). Only subsets of each clustering result are shown, with gene symbols provided to the right of each set of array probes (i.e., each row represents one gene, and each column represents an individual experimental sample).

To assess and compare the global transcriptional profiles of mice with pathological and physiological cardiac hypertrophy, we performed gene expression microarray analysis of RNA from the left ventricles of ISO-treated mice, mice that were exercise trained, and sedentary control animals. The experiments were performed in triplicate, and a separate microarray ran for each individual mouse heart. A gene was considered as differentially expressed if the fold-change was at least 1.5, the Benjamini and Hochberg-corrected (B-H) p value was less than 0.05, and the alteration occurred for each of the 9 possible pairwise comparisons (i.e., each of the 3 controls versus each of the 3 experiments). Based on these criteria, there was a total of 940 probe sets representing 865 different genes (634 up-regulated and 231 down-regulated) that were significantly and reproducibly altered between control and ISO-treated animals. The transcriptional profile of mice that were swam twice daily, on the other hand, was more profoundly affected, with 2,670 probe sets representing 2,534 genes altered (936 up-regulated and 1,598 down-regulated), compared to sedentary control animals. Eight genes were selected for verification by real-time reverse transcriptase polymerase chain reaction (RT-PCR), the results of which validated the microarray analysis (Table 1). The entire list of genes altered in response to exercise or ISO treatment is provided as Additional File 1, and examples are shown in Fig. 5, in which genes with similar expression patterns clustered together using Cluster/Treeview software programs to analyze Z-score transformed signal intensity values from all nine arrays.

**Table 1:** Verification of microarray data by real-time RT-PCR

Gene ID	Gene Symbol	Array		Real-time	
		ISO	Swim	ISO	Swim
		*FC*			
NM_007707	SOCS3	5.9	ND	2.0	ND
M65143	LOX*	7.5	ND	8.0	-2.5
NM_133249	Ppargc1b	-2.8	ND	-1.9	ND
AK018378	Adrb1	-1.7	ND	-1.9	ND
NM_007392	Acta2	-3.7	-3.2	-5.7	-6.5
NM_010809	Mmp3	6.0	ND	3.3	ND
BI78842	Timp4	5.4	3.6	4.9	10.6
AK020831	Adamtsl2	2.0	ND	2.3	ND

Asterisk (*) indicates that a related gene (Loxl1, AF357006) was down-regulated 2.6-fold in exercised mice, compared to sedentary control animals. ND = no significant difference detected. FC = fold-change. Fold-change values shown are representative of 3 independent experiments.

Based on pathway analysis results using Ingenuity Pathway Analysis software, the top associated signalling network functions in mice treated with ISO were cardiovascular disease, free radical scavenging, and molecular transport (28 genes out of 39 network molecules), whereas the top associated network functions for mice that exercised were gene expression, cell morphology, and cell-cell signalling and interaction (35 genes out of 40 network molecules). Interestingly, the most over-represented molecular functions after multiple hypothesis testing correction were cell death for ISO-treated mice (188 molecules, B-H p values 6.1 × 10^-20 ^to 6.6 × 10^-4^) and cellular growth and proliferation for mice that were swam (462 molecules, B-H p values 4.5 × 10^-20 ^to 5.0 × 10^-2^). The top physiological functions in ISO-treated mice were immune system development and function (121 molecules, B-H p values 1.9 × 10^-9 ^to 6.6 × 10^-4^) and immune response (113 molecules, B-H p values 5.7 × 10^-9 ^to 6.6 × 10^-4^), whereas the most over-represented physiological functions in exercised mice were organismal survival (175 molecules, B-H p values 1.0 × 10^-11 ^to 5.0 × 10^-2^) and organ development (146 molecules, B-H p values 2.0 × 10^-6 ^to 1.6 × 10^-3^). Molecular pathway analysis also indicated that cardiovascular system development and function (149 molecules, B-H p values 7.2 × 10^-6 ^to 4.1 × 10^-3^) was a major physiological function associated with genes altered in exercised mice, but not in ISO-treated animals. The top canonical signalling pathways in ISO-treated mice included acute phase response (24 molecules, B-H p value 4.7 × 10^-7^), fibrosis (14 molecules, B-H p value 5.5 × 10^-6^), NRF2-mediated oxidative stress response (16 molecules, B-H p value 1.2 × 10^-2^), and VEGF signalling (10 molecules, B-H p value 1.2 × 10^-2^). The most over-represented signalling pathways identified by Ingenuity for the list of genes altered in response to exercise were PI3K/AKT (37 molecules, B-H p value 8.6 × 10^-9^), ERK/MAPK (44 molecules, B-H p value 9.7 × 10^-7^), integrin (45 molecules, B-H p value 1.7 × 10^-6^), PPAR signalling (27 molecules, B-H p value 1.8 × 10^-6^), and PPARα/RXRα activation (40 molecules, B-H p value 2.3 × 10^-6^).

The vast majority of genes altered in response to ISO treatment or swimming were specific to the condition (that is, there was relatively little overlap in the types of genes altered under these two conditions.) However, there were 159 genes that were altered by both types of cardiac hypertrophy, whose functions were mainly related to immune response, particularly antigen presentation (14 molecules, B-H p values 2.1 × 10^-5 ^to 3.1 × 10^-2^) and hematological system development and function (29 molecules, B-H p values 1.4 × 10^-6 ^to 4.0 × 10^-2^). Of these 159 overlapping genes, 139 were altered in the same direction in pathological and physiological cardiac hypertrophy models (i.e., 42 up-regulated and 97 down-regulated in ISO-treated and exercised trained mice, compared to sedentary control animals). Conversely, there were 20 genes that were altered in each condition in the opposite direction (e.g., up-regulated in ISO-treated mice and down-regulated in exercised animals) (Table 2).

**Table 2:** Genes with overlapping but opposite alteration in ISO-treated and exercise mouse hearts

Gene ID	Gene Name	Gene Symbol	Function	ISO		Swim	
				*FC*	*p*	*FC*	*p*
BE133150	ESTs	NA	Unknown	2.0	0.002	-2.1	0.011
BC019553	cDNA	NA	Unknown	2.6	0.002	-2.9	0.002
BI180630	ESTs	NA	Unknown	2.0	0.007	-2.1	0.007
BM234799	cDNA	NA	Actin cytoskeleton organization	1.7	0.009	-2.0	0.006
BB283973	cDNA	NA	Unknown	-3.1	0.010	2.4	0.020
AW537707	Actin, beta, cytoplasmic	Actb	Non-muscle cytoskeletal constituent	-3.0	0.009	1.8	0.009
NM_007398	Adenosine deaminase	Ada	Smooth muscle contraction; immune response	2.2	0.004	-1.9	0.005
AV229424	Collagen, type V, alpha 2	Col5a2	Extracellular matrix structural constituent	2.2	0.022	-2.2	0.005
NM_010188	Fc receptor, IgG, low affinity III	Fcgr3	Antigen processing and presentation via MHC class I	2.1	0.006	-1.6	0.003
BC004724	Fibronectin 1	Fn1	Acute-phase response; cell-matrix adhesion	2.3	0.020	-1.7	0.005
AK012898	Glycine/arginine rich protein 1	Grrp1	Unknown	3.5	0.005	-2.3	0.005
BB269715	Hypoxia inducible factor 1, alpha subunit	Hif1a	Angiogenesis; neural crest cell migration; response to hypoxia	-1.9	0.002	2.0	0.002
BE995678	Heat shock protein 90, beta (Grp94), member 1	Hsp90b1	Stress response	-8.4	0.009	3.4	0.014
NM_030127	HtrA serine peptidase 3	Htra3	Regulation of cell growth	1.8	0.006	-2.2	0.006
BC003209	Integrator complex subunit 3	Ints3	snRNA processing	2.2	0.006	-2.7	0.006
NM_008606	Matrix metallopeptidase 11	Mmp11	Collagen catabolism	2.3	0.013	-1.9	0.006
NM_010929	Notch gene homolog 4	Notch4	Patterning of blood vessels; cell fate determination	2.0	0.005	-2.0	0.006
AK013448	Protein phosphatase 1, regulatory (inhibitor) subunit 14c	Ppp1r14c	Signal transduction	-2.8	0.002	2.2	0.003
BE137091	Regulatory solute carrier protein, family 1, member 1	Rsc1a1	Regulation of protein transport and modification	2.0	0.005	-1.8	0.006
NM_011581	Thrombospondin 2	Thbs2	Inhibits microvascular endothelial cell proliferation; cell adhesion; wound repair	2.2	0.002	-1.7	0.004

FC = fold-change. Dash ("-") before number indicates down-regulation, compared to appropriate controls. NA - not applicable, where a gene without a gene symbol was identified as altered in expression (i.e., cDNA, EST, or gene encoding a hypothetical protein). ISO and Swim = Control mice versus isoproeteronol-treated or exercised mice, respectively. Gene functions were obtained from Ingenuity pathway analysis software, supplemented with information obtained from the NCBI and Stanford SOURCE search databases.

### Comparison of physiological and pathological cardiac hypertrophy across various studies and models

To compare our results to previously published studies, we obtained all available data files from the GEO website for experiments previously conducted to measure transcriptional responses to exercise-induced cardiac hypertrophy or experimentally-induced pathological hypertrophy. Exercise models included swimming mice and various rat strains trained on treadmills for various time intervals [11-17]. Experimentally-induced pathological cardiac hypertrophy models causing significant cardiac dysfunction and varying degrees of cardiac failure and mortality included familial hypertrophic cardiomyopathy (FHC) in mice (achieved via an R403Q missense mutation in one allele of the α-myosin heavy-chain gene, αMHC^403/+ ^[18]), myocardial infarction in mice [19], aortic banding (i.e., transverse aortic constriction) [20-24] in various strains of rats and mice, arteriovenous shunt, myocardial infarction, and hormone (angiotensin II and 3,5-diidothyroproionic acid) treatments in rats [22], as well as high salt diet in Dahl rats [14]. We also obtained microarray data comparing "normal" human hearts (non-diseased samples obtained during autopsy) to heart samples collected during heart transplant surgeries from patients with end-stage heart failure (idiopathic, ischemic, aortic stenosis, and congenital) [25]. For Affymetrix data in which CEL files were available, data were RMA normalized, Student's t test was performed, and each group was averaged in order to obtain a fold-change value (using GeneSifter software). This less stringent data analysis approach was intended to make the results more comparable across microarray platforms and studies and minimize the level of false negatives. For data provided only in text form, data were assumed to be pre-normalized by the previous researchers (per GEO submission requirements), and Student's t test and fold-change were calculated directly from the provided data. For two of the previous studies [16,17], raw data were not publicly available; we therefore used the gene list provided in the publications. All studies, including overall comparative results, are listed in Table 3.

**Table 3:** Comparison of Multiple Cardiac Hypertrophy Studies

Study	Model	Array	Samples	Time course	n =	Sig Genes	Common Genes	
							Swim	ISO
**Physiological CH Models**								
This Study	C57BL/6 mice	Affy 430 2.0	Control	8 wk	3	2,351	-	139/20
			Swim		3			
GSE77^a ^[11]	FVB mice	Affy U74Av2	Control	10 min	3	141	2/26	5/6
			Swim		3			
			Swim	2.5 day	3	32	10/2	4/3
			Swim	1 wk	3	41	11/3	8/2
			Swim	2 wk	3	77	9/7	10/9
			Swim	3 wk	3	51	2/1	8/3
			Control	4 wk	3	68	14/14	6/5
			Swim		3			
			Control	4 wk/1 wk	3	13	2/1	1/1
			Swim/Rest		3			
GSE739^b ^[12]	Wistar rats	Affy U34A	Sedentary	7 wk	6	39	1/2	7/1
			Treadmill		6			
GSE7640^a ^[13]	Rats [d]	Affy 230 2.0	Sedentary	10 wk	10	18	1/0	1/0
			Mild exercise		10			
GSE776^a ^[14]	Dahl rats	Affy U34A	Sedentary	3 wk	3	293	20/40	7/18
			Treadmill		3			
			Sedentary	6 wk	3	282	12/36	8/14
			Treadmill		3			
GSE9445^a ^[15]	N:NIH LCR rats	Affy 230 2.0	Sedentary	8 wk	4	19	0/1	2/0
			Treadmill		4			
	N:NIH HCR rats		Sedentary		4	42	1/1	2/1
			Treadmill		4			
Diffee *et al*^c ^[16]	Sprague-Dawley rats	Affy U34A	Sedentary	11 wk	9	27	1/1	1/4
			Treadmill		9			
Iemitsu *et al*^c ^[17]		Atlas 3.8I	Sedentary	8 wk	8	75	4/5	1/1
			Treadmill		8			
**Pathological CH Models**								
This Study	C57BL/6 mice	Affy 430 2.0	Control	10 day	3	808	139/20	-
			ISO-treated		3			
Kim *et al*^c ^[31]	FHC Mice [d]	PMAGE	Wild-type	8 wk	4-5	706	215/272	121/79
			αMHC^403/+^	25 wk				
GSE1621^a ^[20]	FVB mice	Affy U74av2	Sham	48 hr	4	15	0/3	4/1
			TAC		5			
			Sham	10 day	4	106	9/30	10/5
			TAC		5			
			Sham	3 wk	4	95	8/21	13/3
			TAC		5			
GSE2459^b ^[21]	FVB mice	Affy U74av2	Sham	10 wk	9	307	14/24	14/7
			TAC		6			
GSE1145^a ^[25]	Humans	Affy 133 2.0	Normal	N/A	11	-	-	-
			Idiopathic dilated		15	2,480	124/200	35/99
			Ischemic		11	1,214	66/74	38/45
		Affy U95A	Normal		4	-	-	-
			Aortic stenosis		7	57	5/4	4/2
			Congenital		6	354	10/50	1/18
GSE738^b ^[22]	Wistar rats	Affy U34A	Sham	3 wk	2	50	1/8	2/1
			Shunt		2			
			Sham	8 wk	2	32	0/5	2/2
			Shunt		2			
			Sham	3 wk	2	55	4/8	12/1
			Myocadial infarction		4			
			Sham	9 wk	2	99	4/15	5/4
			Myocadial infarction		4			
			Sham	6 wk	2	64	6/1	6/1
			Aortic banding		2			
			Sham	12 wk	2	21	2/1	3/0
			Aortic banding		2			
			Sham	16 wk	2	101	5/6	3/2
			Aortic banding		2			
			Sham	30 wk	2	107	5/15	10/3
			Aortic banding		4			
			Vehicle	2 wk	2	-	-	-
			Ang2		2	28	0/1	3/0
			Dipta		2	5	0/1	0/0
GSE5500^a ^[23]	Mice [e]	Affy 430 2.0	Sham	7 day	4	-	23/74	39/10
			TAC		6	470		
GSE12337^a ^[24]	Mice [d]	Affy 430 2.0	Sham	28 day	4	-	0/8	2/0
			TAC		4	12		
GSE775^b ^[19]	Mice [d]	Affy U74Av2	Control	1 wk	4	1,585	69/127	93/29
			Myocardial infarction		3			
			Control	8 wk	4	698	48/34	23/14
			Myocardial infarction		3			
GSE776^a ^[14]	Dahl rats	Affy U34	Control	3 wk	3	-	2/9	8/7
			High salt diet		3	62		
			Control	6 wk	3		40/24	15/6
			High salt diet		3	315		
			Control	15 wk	3	-	37/11	36/3
			High salt diet		3	224		

^a^CEL files analyzed, ^b^Text files analyzed, ^c ^Genes obtained from published article, ^d^strain information not provided by submitters, ^e^C57BL6/J-FVB/N genetic background; Ang2 = Angiotensin II; Dipta = 3,5-diiodothyropropionic acid, FHC = familial hypertrophic cardiomyopathy

As expected, there was a great deal of variation in the total numbers of altered genes between the various studies, array platforms, disease models, strains, and time frames. It is perhaps worth noting, however, that our intention was not to highlight potential differences in experimental technique between groups or compare the potential value of one disease model over another. Rather, we hoped to identify a set of genes that would represent those canonical changes that are captured most frequently under various experimental conditions and thus might represent consistent genetic expression alterations that might be applicable to the study of the human end-stage failing heart. We therefore grouped study types together and considered the most profound alteration (i.e. largest fold-change in magnitude) as representative for that group. Groups were chosen based on model type and animal, while more detailed parameters (e.g., strain, time frame, experimental details) were not considered. This resulted in 14 distinct experimental groups with relevant cardiac expression data: 2 exercise models (swimming mice and rats trained on treadmills), 8 pathological models (mice with aortic banding and myocardial infarction, αMHC^403/+ ^mice, rats with aortic banding, drug-induced cardiac hypertrophy, myocardial infarction, arteriovenous shunt, and high salt diet), and 4 human diseases (idiopathic, ischemic, aortic stenosis, and congenital cardiac hypertrophy).

Based on our analyses and comparison methods, there were 59 genes that were altered in response to exercise in our study and in swimming mice from other studies or treadmill-exercised rats (Table 4). However, there were only two genes (*Cd74 *and *Col3a1*) that were altered in our study and in both mice and rats irrespective of the model type or study. *Col3a1 *was of particular interest, because it was down-regulated in response to exercise in mice (1.6, 3.1-fold) and rats (1.8-fold) but up-regulated in most pathological cardiac hypertrophy models, including αMHC^403/+ ^mice (2.2-fold), aortic binding in mice (3.1-fold) and rats (1.6-fold), arteriovenous shunt (1.7-fold), myocardial infarction (2.5-fold), and idiopathic and ischemic cardiac hypertrophy in humans (1.5- and 2.0-fold, respectively). Interestingly, *Col3a1 *was down-regulated (2.9-fold) in Dahl rats fed a high salt diet, but only at the latest time point tested (15 wk). There were two exercise-induced genes that differed in directionality depending upon which type of rodent or experimental model was used. *Timp3 *was up-regulated in swimming mice but down-regulated in exercised rats. Conversely, *dbp *was up-regulated in rats and down-regulated in mice (Table 4). Interestingly, 8 genes, including dbp, that were represented by more than one "polony" (analogous to an array probe set), were detected as up-regulated and down-regulated by PMAGE in αMHC^403/+ ^mice, compared to wild-type littermates (Table 4). This result could reflect the greater sensitivity of PMAGE to detect transcripts present at low abundance (<1 copy/cell), transcript isoform differences, or detection of technique-specific artifacts resulting from the use of fold-change as a filtering criterion.

**Table 4:** Genes altered in response to exercise, grouped by function

Gene	Physiological			Pathological											
	This study	Swim	Tread	Drug	RQ	MI	AB		Sh	MI	Salt	Idiop	Isch	AS	Cong
	Mice		Rats	Mice				Rats				Humans			
	*FC*														
**Cell adhesion/migration**															
Acta2	-3.2	-1.9		-3.7	1.7			1.8		1.6	-2.6				
Aplp2	-1.7		-2.1		+/-										
Lmo7	1.8		1.7			-8.2						-1.5	-1.5		
Ap2s1	-2		-1.6								1.7				
**Cellular assembly and organization**															
Itga7	-2.8		-1.6								-1.5				
Nfia	1.9		1.9						-1.6		-2.3				
Sorbs1	11.3	2.1			d	-6.7									
Tmsb10	-2.6	-1.5				2.8	2.3				-2.1				
**Cell death**															
Aes	-2.2		-1.9		+/-	-5.2				1.6	1.8				
Akap1	-1.9		-1.7	-2.1	-2.6	-7.3					1.9				
Cd74	-3.8	-2.1	-1.7	-3.1							1.7		1.5		
Cd99	-2.1	-1.6				-3.2									
Crp	2.3		1.7								-2				
Cyb5r3	-2.3		-1.6		i										
Fbn1	-2.8	-1.5				2.9	2.2						1.5		
Gsk3b	6.2		1.6								-1.6	-2.2	-1.7		
H2-D1	-3.3	-1.6													
Kcnk3	-2.6		-2		d	-13.5					2.3		-1.8		
Lamb2	-2.3		-1.7			-3.3									
Mapt	-2.2	-1.7				-15.4						2.4	2.8		
Nfkbia	-2.7	-1.6									1.5	-1.9			
Nme3	-2	-1.5				-3									
Nupr1	-2.5		-1.5		3.3	3.6									
Pitpna	-1.8		-1.5									-1.8			
Ppargc1a	7	2.5				-7.3									
Ppp2r1a	-1.9		-2.9		+/-						2.3				
Tfrc	-4.2		-1.5	-12.3	10.1						1.8	-1.6	-1.7		
**Cell growth and development**															
Dbp	-2.8	-1.6	2.2		+/-			-1.9			-2.4				
Gna12	-2.4	-1.5								1.5					
Ltbp4	-3	-1.7			i		1.6						1.7		
Nedd8	-1.7	-1.6													
Pctk1	-1.7		-1.6		9										
**Heart contraction**															
Dmpk	-3.2	-1.6				-5.2									
MHC-β	11.5	3.4		2.7			4.5	2.6		1.9	2.3	1.6*	2.5*		
**Immune functions**															
C1qa	-2.1	-1.5			3.4	1.7	1.7								
C1qc	-2.4	-1.5				1.7	1.7								
Col1a1	-4.7	-1.6			+/-	3.4	2.4		1.7	2	-2.6	1.6	1.8		1.5
**Col3a1**	**-3.1**	**-1.6**	**-1.8**		**2.2**	**4.9**	**3.1**	**1.6**	**1.7**	**2.5**	**-2.9**	**1.5**	**2**		
Fabp4	2.7		1.7								-1.7				
H2-Aa	-3	-1.6		-3.9		1.9									
H2-K1	-2.9	-1.7			6										
Psmb8	-2.5	-1.6			2.3										
**Extracellular matrix organization**															
Adam19	-2.3	-1.6					1.5								
Col15a1	-3.2	-1.6		-2.8	+/-	2.1	2.3				-2.7				
Matr3	2.1		1.6		1.7										
Timp3	4.5	1.6	-1.5									-1.5			
**Other or unknown**															
Ccdc53	-1.9		-1.5												
Ccdc56	-2.1		-2.1		-2.6						2.2				
Ell2	5	1.8		2.4		-2.2						-1.9	-1.8		
Fstl1	-2	-1.6				6.6	2.8	1.8		1.9	-1.6				
Gas5	-2.5	-1.7										-1.8			
Kank3	-2.1	-1.7													
Lipa	-2.6		-1.7					-1.6							
Ptrf	-2.4	-1.6			+/-										1.5
Slc29a4	1.8	1.7													
Snrp70	2.2		1.6								-1.6				
Ssbp1	-1.7		-1.5												
Tsc22d4	-2.2	-1.5		-1.9	d	-2.9									
Ttc3	2.4		1.6		+/-								1.5		

FC = fold-change. Dash ("-") before number indicates down-regulation, compared to appropriate controls. A blank indicates that no significant alteration (fold change at least 1.5, p value ≤ 0.05) was observed. The asterisk indicates that a variant of MHC-β (MYH7B) was up-regulated. For PMAGE data, "i" and "d" indicate induction and down-regulation with 0 transcripts detected in either wild-type or αMHC^403/+ ^mice, respectively. Where genes that were represented by more than one polony (analogous to a probe set) conflicted in direction (one or more up-regulated sequence tags and one or down-regulated sequence tags representing the same gene), "+/-" is used in place of a FC value. Gene highlighted in bold font was a consistent finding (down-regulated in exercised rodents and up-regulated in various pathological cardiac hypertrophy conditions). Idiop, Isch, AS, and Cong = idiopathic, ischemic, aortic stenosis, and congenital, respectively. "Drug" refers to isoproterenol, angiotensin II, or 3,5-diiodothyropropionic acid treatment. Sh, MI, and AB = shunt, myocardial infarction, and aortic banding, respectively. RQ = αMHC^403/+ ^mice, representing familial cardiac hypertrophy. Gene functions were obtained from Ingenuity pathway analysis software, supplemented with information obtained from the NCBI and Stanford SOURCE search databases. Some genes perform more than one function, but are only listed under one category.

Consistent with molecular signalling events known to occur under conditions of physiological cardiac hypertrophy, there were three PI3K/AKT signalling pathway molecules (*Gsk3b*, *Nfkbia*, and *Ppp2r1a*) altered in exercised mice and rats. Moreover, the PI3K/AKT was statistically over-represented in the 57 genes consistently altered across experimental models, based on Ingenuity pathway analysis (B-H p value 2.2 × 10^-3^). Over-represented cellular processes included cell death (18 molecules, mainly down-regulated, B-H p values 2.2 × 10^-5 ^to 1.8 × 10^-2^), cellular assembly and organization (14 molecules, B-H p values 3.8 × 10^-5 ^to 1.8 × 10^-2^), molecular transport (14 molecules, B-H p values 1.7 × 10^-4 ^to 1.7 × 10^-2^), and cellular development (13 molecules, B-H p values 3.5 × 10^-4 ^to 1.8 × 10^-2^). Immune response (10 molecules, B-H p values 1.7 × 10^-4 ^to 1.8 × 10^-2^) was the most significantly over-represented physiological function.

We used a similar approach to examine ISO-induced transcriptional alterations, expecting that each gene was similarly altered for at least one human disease equivalent to be considered significant. The results of this comparison, including fold-changes obtained from other animal models and also exercised rodents, are shown in Table 5. There were 68 gene expression alterations (36 up-regulated and 32 down-regulated genes) that showed considerable overlap between model types and studies that were also similarly altered in at least one of the four human diseases. This included two traditional markers of cardiac hypertrophy (MHC-β and Atp1a1/SERCA). Ingenuity pathway analysis confirmed that the most over-represented molecular pathways in this canonical list of genes were cellular growth and proliferation (22 genes, B-H p values 1.8 × 10^-7 ^to 6.3 × 10^-3^), cell-cell signalling and interaction (14 genes, B-H p values 2.4 × 10^-7 ^to 6.3 × 10^-3^), cell death (20 genes, B-H p values 7.4 × 10^-7 ^to 6.3 × 10^-3^), cellular movement (14 genes, B-H p values 9.9 × 10^-7 ^to 6.3 × 10^-3^), and cellular development (23 genes, B-H p values 1.3 × 10^-6 ^to 6.3 × 10^-3^). Twelve of the genes were specifically associated with cardiovascular system development and function (B-H p values 1.2 × 10^-6 ^to 6.3 × 10^-3^).

**Table 5:** Genes altered under conditions of pathological cardiac hypertrophy, grouped by function

Gene	Pathological													Phys
	This Study	RQ	MI	AB		Sh	MI	Drug	Salt	Idiop	Isch	AS	Cong	Exercise
	Mice				Rats					Humans				Rodents
	*FC*													
**Cell adhesion and migration**														
Aoc3	2.2									1.5				
Itgbl1	2.4			1.8						1.6	2.8			
Ltbp2	3.3		13.1	2						1.7	2.5			
Omd	2.6		5.1							1.9	2.6			2.4
**Cardiovascular functions and/or disease association**														
Angptl2	-2.2			1.7						-1.5				-2.3
Atp1a1	-1.9	d	-4.0				1.8		2.3	-1.7	-1.6			-5.3
Ctgf	3.7	2.1	3.1	2.7							1.5	2.2		-3.5
Dcn	2.8	1.8					1.6		1.7		1.6			-1.6
Fbxw7	-1.8									-1.5				
Fn1	2.3	1.9	1.8	3	1.7		1.9		1.6	-1.6	-1.6	2		2
Hif1a	-1.9			1.5						-1.9				-1.5
Hyou1	-1.8								-1.9	-1.7				
Kdr	-1.9	7							-1.7	-1.5				2.3
Lox	10.1		24.6	3.5					1.5		2			
Mef2c	-2.2		-3.1							-1.6				
MHC-β	2.7			4.5	2.6		1.9		2.3	1.6*	2.5*			
Prox1	-1.8		-20	-1.5						-1.8	-1.6			
Sfrs2	-2.7												-1.7	3.4
Thbs2	2.5		7								1.8			
**Cell-cell signaling**														
Palld	-3.4									-1.9				2
Ramp1	-3										-1.6			
**Cellular Movement**														
Trak1	-2									-1.6				
Tuba4a	-6		-7.7						-2.8		-1.7			
Tubb2a	-2			1.6						-1.6				
**Cell cycle arrest and/or cell death**														
Ddit4	7											1.9		-2.2
Il17rd	1.9										1.6			
Isg20l1	-1.6									-1.6				-3.6
Prmt1	-2.1									-1.8				
Scn7a	2.1				-2.1						1.9			
**Development**														
Ezh1	1.9										1.8			
Hopx	-2.5		-18.5							-2.5	-3.1			
Slc40a1	2.1								1.5	1.6	2.1			-1.7
Ttll7	4.2									1.7				
**Cell growth/proliferation**														
Brd4	-1.8									-1.6				
Ccng2	3	-1.7									1.8			
Cdh13	-8.3	d	-6.7										-2	
Clu	2.1	2.7		1.7	1.5		1.6		1.8			1.5		-1.5
Cthrc1	3.5			2.5							2.4			
Egr1	-3.3			1.9	-1.7	1.8			1.6	-2.2	1.9			-2.8
Fkbp4	-2.6		-7.2		-1.6				-2.1	-1.6	-1.5			
Hipk2	-2	4								-1.5	-1.6			-1.6
Hk2	-2		-6.9						-1.8	-1.7				1.5
Hsp90b1	-8.4	-1.6								-1.6				
Kif1b	-3.6	3	-3.6							-1.6				
Mlf1	-2	-2.1	-25.2										-1.5	-2
Nox4	4.7										1.6			
Pdgfd	1.9								-1.6		2.1			
Plagl1	2.4		3.5								1.8			-4.2
Plcb4	1.8										1.6			
Postn	4.1	3.1	5.8	7.1	4.8		3.9	2			2.8			-2.2
Tfrc	-12.3	10							1.8	-1.6	-1.7			-1.5
Trp53inp1	4.3										1.5			
**Extracellular matrix morphology**														
Aspn	2.6	1.6		3	2		1.8			2.6	4.5			
Cilp	3.8	5.4		2.8							1.9			
Fbln2	1.7	1.5	3.2	2.1						1.6	1.6			2.2
Mfap5	3.1	1.5	3.8	2.9							1.8			-4.2
**Other or unknown**														
Arrdc3	2.1										1.8			
Dhrs7	-1.6									-1.5				
Hs2st1	-2		1.8							-1.8				
Kcnt2	2.2									-1.9	1.6			
Klhl24	2.7		-3.1								1.6			-1.6
Obfc2a	-2.1									-1.8				-1.7
Pcmtd2	2.1					-1.8					1.6			-3
Reep1	-2.8		-3.9							-2.5	-1.7			
Rnase4	2		2.6						1.5		1.9			
Trmt5	2.4	i									1.5			
Tsfm	-1.7		-3.6							-1.5				
Zbtb44	2									-1.6	1.6			
Zfp428	-2.2												1.5	

FC = fold-change. Dash ("-") before number indicates down-regulation, compared to appropriate controls. A blank indicates that no significant alteration (fold change at least 1.5, p value ≤ 0.05) was observed. The asterisk indicates that a variant of MHC-β (MYH7B) was up-regulated. For PMAGE data, "i" and "d" indicate induction and down-regulation with 0 transcripts detected in either wild-type or αMHC^403/+ ^mice, respectively. Idiop, Isch, AS, and Cong = idiopathic, ischemic, aortic stenosis, and congenital, respectively. "Drug" refers to angiotensin II treatment. (No gene alterations in common with ISO treatment were found for 3,5-diiodothyropropionic acid treated-rats.) Sh, MI, and AB = shunt, myocardial infarction, and aortic banding, respectively. RQ = αMHC^403/+ ^mice, representing familial cardiac hypertrophy. Phys = physiological. Gene functions were obtained from Ingenuity pathway analysis software, supplemented with information obtained from the NCBI and Stanford SOURCE search databases. Some genes perform more than one function, but are only listed under one category.

### Analysis and comparison of animal cardiac hypertrophy models to human cardiac disease

To identify genes that are most likely to be relevant to human disease, we compared the microarray results from our study along with all the previous studies listed in Table 3 to the GSE1145 gene expression dataset (i.e., human idiopathic, ischemic, aortic stenosis, and congenital cardiac hypertrophy). An average fold-change was calculated to represent each model type (i.e. cardiac hypertrophy induced by drug treatment, aortic banding, myocardial infarction, shunt, high salt diet, or exercise). Inclusion in the final list of relevant genes required that at least one of the rodent pathological cardiac hypertrophy models (i.e., drug, aortic banding, myocardial infarction, shunt, or salt) exhibit an alteration in the same gene in the same direction. Also included were genes that were altered in the opposite direction (i.e., up-regulated in humans and down-regulated in animals) if the alteration was observed in at least two animal models. Finally, to eliminate those genes that might be specific to just one group of humans (or one type of cardiac hypertrophy) an alteration was expected to have occurred in at least two out of the four human diseases included.

**Figure 6:**
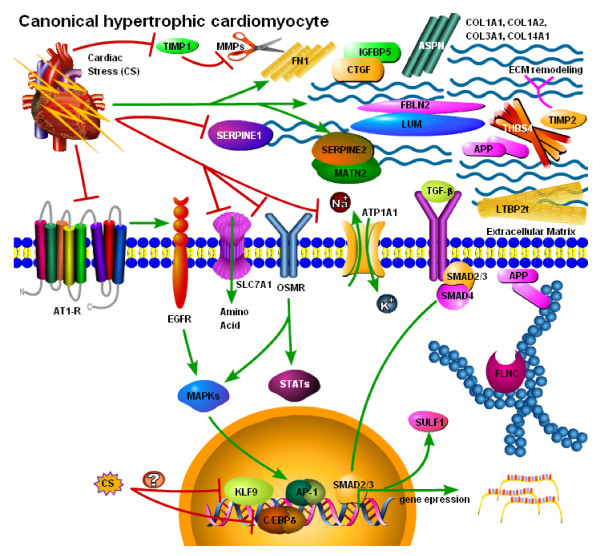
**Connective tissue associated signalling network common to various cardiac hypertrophy animal models and human cardiac disease**. Ingenuity Pathway Analysis software was used to identify a signalling network associated with connective tissue disease, overlaid with average expression data common to various animal models of cardiac hypertrophy and human cardiac diseases. Pathway Builder was then used to create a signalling pathway, based on the Ingenuity signalling network, Biocarta's pathway database, and literature-based associations. TGF-β, which was not identified using microarrays but might nonetheless play a non-transcriptional role in heart disease, is shown as a plausible commonly activated pathway in the human failing heart and various animal models of cardiac hypertrophy. Also shown are the various extracellular matrix proteins that were transcriptionally up-regulated in animal models and human disease, based on microarrays. Signalling pathways associated with commonly down-regulated transcripts (e.g., AT1-R and EGFR) are also shown.

When examined individually, there were 140, 134, 19, and 35 genes in humans with idiopathic, ischemic, aortic stenosis, and congenital-related cardiac hypertrophy, respectively, that exhibited significant overlap with results obtained from studies using animal models (data not shown). When the four lists were compared, there were 64 genes that were significantly altered in at least two of the human diseases that also exhibited significant overlap (based on the aforementioned criteria) with the animal models (Table 6). Interestingly, The most statistically significant over-represented molecular and cellular functions, based on Ingenuity Pathway Analysis software, were cell death (23 genes, B-H p values 1.2 × 10^-7 ^to 5.1 × 10^-3^), cellular movement (22 genes, B-H p values 3.2 × 10^-7 ^to 5.1 × 10^-3^), cellular growth and proliferation (30 genes, B-H p values 1.1 × 10^-6 ^to 5.1 × 10^-3^), cell-to-cell signalling and interaction (21 genes, b-H p values 8.4 × 10^-6 ^to 5.1 × 10^-3^), and cell cycle (15 genes, B-H p values 9.3 × 10^-6 ^to 5.1 × 10^-3^). The most over-represented physiological functions were tissue morphology (18 genes, B-H p values 1.1 × 10^-5 ^to 5.1 × 10^-3^), cardiovascular system development and function (13 genes, B-H p values 1.4 × 10^-5 ^to 5.1 × 10^-3^), connective tissue development and function (10 genes, B-H p values 1.4 × 10^-5 ^to 5.1 × 10^-3^), skeletal and muscular system development and function (16 genes, B-H p values 1.8 × 10^-5 ^to 5.1 × 10^-3^), and organismal development (9 genes, B-H p values 2.6 × 10^-5 ^to 5.1 × 10^-3^). The most profoundly affected signalling network, involving 23 out of the 64 genes broadly associated with animal cardiac hypertrophy models and the human disease indicated that TGF-β in particular might be generally involved in the dysfunctional heart (Fig. 6).

**Table 6:** Genes altered in human pathological cardiac hypertrophy that are recapitulated in animal models

Gene	Human				Animal Models						
	AS	Cong	Idiop	Isch	Drug	RQ	AB	MI	Sh	Salt	Exercise
	**Pathological**										**Phys**
**Adhesion and migration**											
ITGBL1			1.9	2.7	2.4		1.8				
LTBP2			1.6	2.4	3.3		2.0	13.0			
OMD			1.8	2.4	2.6			5.1			
SERPINE2	2.6			1.5		1.8	1.7	2.8			
THBS4	1.7			2.2		2.8	1.9	1.8		1.5	
**Cardiovascular functions**											
AOC3			1.5	1.6	2.2						
APP			2.0	1.9		3.6	1.5			1.7	-1.6
ATP1A1			-1.7	-1.6	-1.9	d		+/-		2.3	-5.3
CCL2			-2.9	-2.7	3.7		1.9	3.7			-2.3
CTGF	2.2			1.5	3.7	2.0	2.4	3.1			-3.6
EGFR			-1.8	-1.5	2.9		-2.2				
FN1	1.9		-1.6	-1.6	2.3	1.9	2.3	1.9		1.6	-1.7
M GCLM			-2.1	-1.6	3.0			1.6			
GSK3B			-2.2	-1.7						-1.6	1.8
SERPINE1			-3.4	-3.6	1.6		1.8	3.4		1.9	
SLC7A1			-2.0	-1.8			-1.6	-6.0			
TGM2			-1.7	-1.7	1.9		1.5				-2.1
TIMP1			-1.9	-1.6	4.8	3.8	2.3	5.4		1.5	1.5
TIMP2			1.6	1.9			1.7	1.5			2.3
**Cell growth/proliferation and survival**											
ACOX1		-1.6		2.1			-1.7	-5.5		1.7	-1.7
AMD1		-1.6	-2.0	-1.6		-2.6	-1.6	-1.6			
CTSC			-2.4	-2.1		-2.0	5.7	4.0		2.0	-1.8
DDX3X		-1.6	-1.6				-2.6				2.5
KLF9		-2.0	-1.7					-3.9		-2.3	-1.6
OSMR			-1.9	-1.5	3.6		1.5				
PAPSS2			-1.8	-1.6	1.8		-1.5				1.5
PXDN			1.6	2.2		1.8	1.7				
SULF1			1.7	2.2			1.9				
TFRC			-1.6	-1.7	-12.3	10.0				1.7	-2.8
**Extracellular matrix morphology**											
ASPN			2.2	4.1	2.6	1.6	2.4	1.8			
COL14A1			2.4	4.5			3.0	6.6			
COL3A1			1.5	2.1		2.1	2.2	3.5	1.7	-4.5	-2.2
FBLN2			1.6	1.6	1.7	1.5	1.9	3.2			
LUM			2.9	3.0			1.9	1.7		-2.2	
MATN2			1.8	2.6			2.1				
**Immune response/modulation, inflammation, and stress response**											
DNAJA4			-2.4	-2.2			1.6	2.0		2.6	-2.9
FKBP4			-1.6	-1.5	-2.6		-1.6	-7.2		-2.0	-2.0
IFIT2			1.6	1.7		2.7	1.7				
IFIT3			1.7	1.8			1.7	1.9			
MGST1			-2.3	-2.4	2.0					2.0	
**Skeletal and muscular functions**											
CEBPD			-2.4	-2.0	6.7		1.5	2.2			-2.0
COL1A1		1.5	1.7	2.1		+/-	2.6	2.6	1.7	-2.4	-1.5
COL1A2	1.9		1.7	2.1		2.7	2.5	3.1		-2.1	-2.2
FRZB			2.1	2.7		i	1.8	9.0			
HSPB6	2.3	1.9								2.0	
IGFBP5	1.6		2.0	2.3		4.5	1.5				-0.5
KBTBD10			-2.3	-1.8			1.8			1.8	
**Tissue morphology**											
BCL6		-2.2	-1.9			+/-	-1.6				
CFH			2.0	2.8	2.8	1.6	1.8	3.2			
EGR1			-2.2	1.9	-3.3		0.1		1.8	1.6	-3.6
FLNC		1.5	-2.1	-1.8			1.6			1.6	-2.7
PRNP		-2.0	-1.6				1.7			2.2	
TXNRD1		-1.5	-2.0		1.6					1.9	-1.8
**Other or Unknown**											
ART3		-1.8	-1.6				-1.6	-9.5			1.7
CCDC80			1.6	2.1	2.1		1.7	2.8			-6.3
FNDC1			2.0	3.4			2.2	5.0			
HMGB2			1.6	1.6			2.1	3.0			
ITIH5			1.6	2.0			2.2				
KCND3		-1.8	-1.7							-1.7	
LDB3			1.6	1.7		-1.9		11.0		1.6	1.8
LYPLA1		-2.1	-1.8				-2.9				0.3
SLC40A1			1.6	2.1	2.1					1.5	-1.7
SYNPO2L			1.6	2.0			2.2				
TAF9B			-1.7	-1.5			-1.7		2.0		

FC = fold-change. Dash ("-") before number indicates down-regulation, compared to appropriate controls. A blank indicates that no significant alteration (fold change at least 1.5, p value ≤ 0.05) was observed. For PMAGE data, "i" and "d" indicate induction and down-regulation with 0 transcripts detected in either wild-type or αMHC^403/+ ^mice, respectively. Where genes that were represented by more than one polony (analogous to a probe set) conflicted in direction (one or more up-regulated sequence tags and one or down-regulated sequence tags representing the same gene), "+/-" is used in place of a FC value. Idiop, Isch, AS, and Cong = idiopathic, ischemic, aortic stenosis, and congenital, respectively. "Drug" refers to angiotensin II, 3,5-diiodothyropropionic acid, or isoproterenol treatment. Sh, MI, and AB = shunt, myocardial infarction, and aortic banding, respectively. RQ = αMHC^403/+ ^mice, representing familial cardiac hypertrophy. Phys = physiological. Gene functions were obtained from Ingenuity pathway analysis software, supplemented with information obtained from the NCBI and Stanford SOURCE search databases. Some genes perform more than one function, but are only listed under one category.

**Figure 7:**
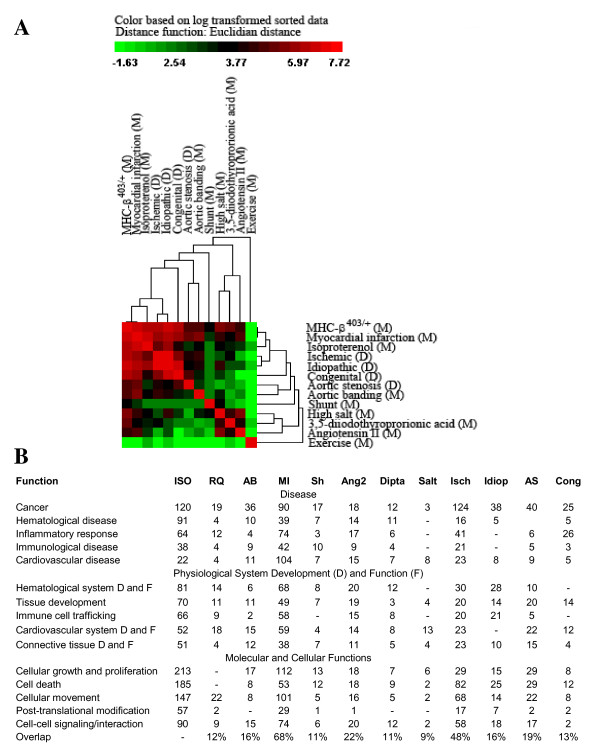
**Hierarchical cluster of a pairwise similarity matrix and ontological analysis to identify relatedness between animal models and human cardiac disease**. A similarity matrix was produced for each model or human disease type, with each pairwise comparison score calculated as the number of genes altered in the same direction (up-regulated or down-regulated at least 1.5-fold on average) compared to the appropriate study controls. The resulting scores were log transformed and Z scores calculated before performing hierarchical clustering using CLUSFAVOR 6.0 software (**A**). Bright red represents the highest pairwise similarity scores, black represents median values, and bright green represents the lowest pairwise similarity values. Each gene list was also analyzed using Ingenuity Pathway Analysis software and function categories for each animal model and human heart failure type compared (**B**). Functional categories shown were significantly over-represented (B-H multiple hypothesis correction p value ≤ 0.05), and numbers represent the number of altered genes in that category.

To compare animal models and human disease data sets, we generated a similarity matrix based on average fold-change observed for each disease type compared to its appropriate study control for all overlapping genes (i.e., genes represented on all array platforms for both rodents and humans). The two data sets for which raw data were unavailable were excluded. Similarity was measured as the number of genes in common with fold-changes that occurred in the same direction (e.g., up-regulated compared to control samples) with a magnitude of at least 1.5. All possible pairwise comparisons were performed, and the resulting matrix was used to create a clustering diagram to illustrate which groups most closely resembled one another (Fig. 7A). As expected, the rodent exercise model least resembled induction of pathological cardiac hypertrophy in animals or human cardiac disease. The Angiotensin II, 3,5-diiodothyropropionic acid, and high salt animal models least resembled human disease, and ISO treatment was most similar to ischemic and idiopathic cardiac failure in humans. Similar results were obtained when ontological categories of altered genes were analyzed for each model and disease group using Ingenuity Pathway Analysis software program (Fig. 7B). As shown, the most common functions were related to cellular growth and proliferation, immune-related signalling/processes, and cardiovascular functions.

## Discussion

A variety of methods have been used to simulate human heart disease in animals, and a great many studies have been conducted to examine the transcriptional profiles of these animals, especially in conjunction with signalling pathway perturbation or drug discovery. Few studies, however, have attempted to coalesce these various data to identify canonical molecular signalling pathways. A notable exception is Strom et al [22], who comprehensively compared several different rodent models of cardiac hypertrophy to identify a core set of genes that might generally characterize cardiac hypertrophy signalling. This previous study, however, did not include ISO, which is thought to induce myocardial damage via an increase in catecholamines and subsequent imbalance of energy supply and cardiac hyperactivity [26]. ISO is mainly used as a cardiac hypertrophy model because it is convenient and yields rapid and reproducible results, but very little is known regarding the precise mechanism of action of this drug or exactly how it induces cardiomyopathy [7]. Our laboratory previously tested an FDA-approved generic antibiotic using the ISO model and found that physiological and microarray data supported the use of doxycycline as an efficacious treatment for ISO-induced cardiac hypertrophy [27]. These results were reproducible using transverse aortic banding and post-myocardial infarction hypertrophic animal models [27]; however, it is nonetheless important to identify the underlying molecular mechanisms of ISO-induced hypertrophy and correlate this animal model with human cardiac disease.

The results of this study show that ISO induces transcriptional alterations in mouse hearts that differ drastically from those elicited by normal hypertrophy in response to exercise training (Additional File 1). Moreover, the differential functions and pathways associated with gene expression alterations elicited by the two types of treatments are consistent with what is known about molecular signalling events that are specific to pathological versus physiological cardiac hypertrophy. For instance, ISO treatment results in the alteration of several genes involved in acute phase and oxidative stress response signalling, which is associated with maladaptive cardiac remodeling [4,5]. Conversely, swimming-induced gene expression alterations in mouse hearts were mainly those known to be involved in normal increases in heart mass resulting from exercise (e.g., PI3K/AKT and ERK MAPK signalling pathways) [2].

Based on our comparisons of our genetic expression results and the results of others using a wide variety of other animal models of cardiac hypertrophy, we conclude that the transcriptional profile of ISO-treated mice most resembles gene expression alterations induced by the transverse aortic banding model. However, the similarity is mainly limited to mice, with few overlapping genes detected between ISO-treated mice and the various rat models. A more extensive study would be required to compare mouse and rat models in order to conclusively determine which best mimics human cardiac disease. Our results indicate that profound differences in gene expression patterns exist between the two types of models that warrant further investigation.

Despite the expected variability between studies arising from differences in animal disease models, strains, experimental variations, array platforms, and species-related differences, our results indicate that among human cardiac diseases, ischemic-related cardiac hypertrophy most closely resembles ISO-treatment of mice (Fig. 7). The similarity of ISO-treatment to idiopathic heart disease suggests that the majority of idiopathic patients exhibited ischemia-associated cardiac hypertrophy and also implies that molecular signatures might be used to classify idiopathic diseases. Alternatively, our findings may simply reflect the notion that a common molecular signature underlies many forms of endstage cardiac failure, irrespective of the etiology. A further complication is that some of the changes in transcript expression may be secondary to changes in cellular composition of cardiac tissue. However, we identified a core set of genes that characterize animal models and human cardiac disease, particularly ischemia-induced cardiomyopathy (Table 6). This list represented several major cellular functions, including cell adhesion and migration, cardiovascular-specific and muscle-specific functions (e.g., cardiac tissue remodelling), cellular growth, proliferation, and survival, extracellular matrix morphology, immune response and inflammation, and stress response.

One potential weakness in comparing human to animal models is that the human samples may have been subjected to varying degrees of warm ischemia during collection and processing, whereas the animal models were probably collected under more controlled circumstances. There are nonetheless some interesting findings in the results obtained from comparing the various animal model and human cardiac disease gene expression profiles, as shown in Fig. 7. First, as might be expected, the genetic expression profile from murine exercised hearts is strikingly dissimilar from any of the expression profiles from either the human diseased hearts or hearts obtained from murine models of human disease. Secondly, the murine cardiac disease models that do not have a readily recognizable human disease analog (that is, animals treated with high salt, 3,5-diiodothyropropionic acid, and angiotensin II) cluster together, and are not particularly similar to the human disease models. Finally, the murine expression profile for animals treated with aortic banding is very similar to the one obtained from humans with aortic stenosis; these physiologic processes would be expected to be very similar, and the extreme similarity in the gene expression profiles for these conditions strongly supports the notion that microarray genetic profile analysis can be used to determine the faithfulness of animal models in replicating human disease.

## Conclusion

We characterized the transcription profile of mouse hearts exposed to isoproterenol, a convenient, reproducible, and rapid model of cardiac hypertrophy. We also indentified the animal model and human cardiac disease transcriptomes that most closely resemble ISO-treatment: myocardial infarction and mutation of αMHC in mice and ischemia-induced cadiomyopathy. We further identified a core set of genes that might represent a universal maladaptive cardiac response to stress and provide individual gene candidates for further investigation.

## Methods

### Animal models of cardiac hypertrophy

Eight-week old male C57BL/6 mice were purchased from the Jackson Laboratory and housed in the Animal facility at University of Texas Southwestern Medical Center (UTSW), Dallas, TX, in accordance with the standards set forth in the *Guide for the Care and Use of Laboratory Animals (NIH Publication No. 85-23, revised 1996)*. All experimental procedures for this study were approved by the Institutional Animal Care and Use Committee at UTSW. Pathological cardiac hypertrophy was investigated using the isoproterenol-induced subacute myocardial injury model as previously described [28]. Briefly, animals were anesthetized with 1.5% isoflurane (Smiths Medical PM, Waukesha, WI) in 98.5% oxygen and a 1 cm incision made on the back of each animal between the shoulder blades. An Alzet 1007D micro-osmotic pump (DURECT Corporation, Cupertino, CA) containing isoproterenol (ISO, Sigma-Aldrich, St. Louis, MO), at 40 mg.kg^-1^.d^-1^, dissolved in 0.9% NaCl was inserted into the infrascapular subcutaneous tissue and the incision sutured. After 10 days of ISO administration, mice were sacrificed, and left ventricles were collected and processed for subsequent assays.

Experimental physiological cardiac hypertrophy was induced via exercise, as previously described [29]. Briefly, mice were placed in buckets of pre-warmed water maintained at ~30°C with low-watt heat lamps and allowed to swim for 90 minutes twice daily. At the beginning of the experiment, mice were acclimated to the exercise routine gradually, beginning with 10 minutes twice daily and increasing in increments of 10 minutes per day, until 90 minutes was obtained. After 8 weeks of swimming, mice were sacrificed and heart samples (left ventricles unless otherwise stated) collected and processed for each assay. Sedentary mice confined to cages served as negative controls.

### Histology

Hearts were excised from mice following euthanasia, grossly trimmed in frontal orientation, blotted free of excess blood and embedded in Tissue Freezing Medium (Triangle Biomedical Sciences, Durham, NC). Rapid freezing of embeds was achieved by partial immersion in liquid-nitrogen-supercooled isopentane prior to storage at -80°C. At a later time, embeds were equilibrated to -24°C and sectioned on a Leica CM3000 cryostat (Wetzlar, Germany). Eight-micron slices were adhered to silanated slide glass (Superfrost Plus, Fisher Scientific, Pittsburg, PA) and air-dried. Resulting sections were stained with routine hematoxylin and eosin (H&E) for histopathological analysis or with Masson trichrome to assess fibrosis. In order to assess apoptosis, TUNEL (TdT-mediated dUTP nick end labeling) assays were performed using the DeadEnd Fluorometric TUNEL System, as described by the manufacturer (Promega Corporation, Madison, WI). Specimens were photomicrographed in bright field on a Leica DM2000 microscope (Wetzlar, Germany), equipped with an Optronics Microfire CCD camera (Goleta, CA). Image acquisition was conducted using Optronics PictureFrame 2.0 software (Goleta, CA) and morphometric analysis was conducted using Image J 1.38w software (NIH, Bethesda, MD) for Macintosh computers.

### Microarrays

Microarray sample preparation and analysis was performed as previously described. Briefly, total RNA from left ventricles of experimental animals was isolated using TRIzol Reagent (Invitrogen Corporation, Carlsbad, CA) per manufacturer's instructions and purified by phenol-chloroform extraction and ethanol precipitation. RNA (20 μg) was further processed and hybridized to the GeneChip Mouse Genome 430 2.0 Array (Affymetrix, Santa Clara, CA) by the Microarray Core Facility at University of Texas Southwestern Medical Center per manufacturer's instructions. Data were analyzed using GeneSifter (VizX Labs, Seattle, WA) and Spotfire DecisionSite 9.0 (Spotfire, Inc., Somerville, MA). Briefly, data were normalized using robust-multi average (RMA) method, and signals for each group were averaged before performing Student's t test with Benjamini and Hochberg correction and pairwise comparisons for sedentary mice versus mice that received ISO treatment or were exercised. Genes were considered as altered if the folds-change was at least 1.5 and adjusted p value ≤ 0.05. Consistency was also expected, which was assessed by performing all possible pairwise comparisons of individual samples. A fold-change of at least 1.5 was expected for each replicate comparison (e.g., sedentary mouse 1 versus swimming mouse 3), and this alteration was expected to be at least 50% greater than the fold-change derived from comparison of any two replicate samples (e.g., sedentary mouse 1 versus sedentary mouse 2). This effectively eliminated differences that might arise as a result of natural biological variation between mice. Raw and processed data (a total of 9 arrays) were deposited in the Gene Expression Omnibus (GEO) online http://www.ncbi.nlm.nih.gov/geo database (Accession GSE18801).

For comparison of our gene expression results to previous studies, raw microarray data (CEL or text files) from mice or rats with pathological or physiological cardiac hypertrophy induced by a variety of methods (details provided in Table 3), were downloaded from Gene Expression Omnibus http://www.ncbi.nlm.nih.gov/geo/. Gene expression data from autopsied human hearts and failing hearts collected during heart transplant surgeries were also obtained from Cardiogenomics PGA [19]. Raw data were RMA normalized using GeneSifter (or assumed to already be normalized if only text files were provided) and Student's t test and pairwise comparisons subsequently performed using the appropriate included controls for each data set. A fold-change of 1.5 or greater and p value ≤ 0.05 were considered as altered. The results from two additional studies [16,17] for which raw array data were unavailable were also included, using the reported gene lists provided in the publication or as supplementary material. Likewise, supplementary pre-processed data (i.e., analyzed by original study researchers) from a study using a novel approach (polony multiplex analysis of gene expression, or PMAGE), which is designed to achieve a much higher level of sensitivity than traditional gene expression microarrays, was included. PMAGE data were filtered based on average fold-difference (= 1.5) in order to make the data comparable to microarray-based results. However, only 10 potentially relevant genes were excluded due to this additional criterion.

To link our study results (i.e., gene expression profiles of ISO-treated and exercised mice) to results obtained from the various other animal and human studies, a perl script was written to match genes across the various lists by Accession number (for same species results) or gene symbol (for inter-species results comparisons). If neither the Accession number nor gene symbol matched perfectly, then the gene descriptions were compared and same identify was considered if the names differed by nor more than 25%. If the ISO or swim results contained more than one match for a single data point in a published study, only the top three matches were recorded, with the best match ordered first. This perl script was later modified to compute averages for the multiple matches. This method would be expected to fail to indentify some homologous genes in which there are name designation differences between species (e.g., the mouse version of human IL-8 is referred to as KC or GRO). However, our intention was to greatly minimize false positive and thus compare only those genes that were truly the same between the various data sets. To ensure that this was indeed the case, all final genes lists representing overlap between study results were also manually examined and any genes that were computationally misidentified subsequently removed.

### Similarity matrix

To produce a similarity matrix, average fold change was calculated for all animal model and human disease samples, compared to its appropriate control after normalization (e.g. ischemic heart compared to non-diseased autopsied heart). The two studies for which raw data were unavailable were excluded (all samples listed in Table 3). Statistical tests were not applied, but an alteration was only considered if the magnitude of change was at least 1.5-fold. A perl script was written to combine the resulting data for the ~2,300 genes that were represented on all arrays using the official gene symbol as the criteria for matching the genes across platforms and species. For genes represented more than once on an array, the alteration with the highest magnitude was recorded. The fold-changes for the various similar sample sets (e.g., exercised rodents) were averaged and a simple similarity scoring scheme applied based on the numbers of genes with same-direction alteration (fold change at least 1.5) between each pairwise disease condition comparison. These scores were then log transformed and converted to Z scores and the resulting values clustered.

### Hierarchical clustering

Hierarchical clustering of genes was performed using Cluster/Treeview (Eisenlab, http://rana.lbl.gov/eisen/). For comparison for the two cardiac hypertrophy models used in this study (ISO-treated and swimming mice compared to sedentary controls), normalized signal values were used for clustering after Z score transformation. The similarity matrix was clustered using normalized pairwise scores as described above using CLUSFAVOR 6.0 (Baylor College of Medicine, Houston, TX).

### Real-time reverse transcriptase polymerase chain reaction (RT-PCR)

Real-time reverse transcriptase (RT)-polymerase chain reaction (PCR) was performed in the iCycler iQ (Bio-Rad, Hercules, CA) using SYBR Green I dye (QIAGEN, Valencia, CA), as previously described [27]. Briefly, each 25-μl reaction contained 100 ng of RNA, 2.5 μl of primers (Quantitect Primer Assays; QIAGEN), 12.5 μlof SYBR Green PCR master mix and 0.25 μl of reverse transcriptase. Negative controls containing water instead of RNA were concomitantly run to confirm that the samples were not cross-contaminated. Targets were normalized to reactions performed using Quantitect GAPDH primers (QIAGEN), and fold change was determined using the comparative threshold method [30].

### Statistical Analysis

Values presented are expressed as mean ± S.E.M. All comparisons between groups were performed using a one-tailed Student's *t *test or ANOVA. Differences were considered statistically significant for *p *< 0.05.

## Authors' contributions

CLG oversaw the project, carried out the microarray analysis and subsequent interpretation of data, participated in the mouse, histology, and real-time RT-PCR experiments, and drafted the manuscript. MAS aided in experimental design, data interpretation, and manuscript preparation and also provided medical expertise. ME carried out the initial animal experiments and participated in subsequent sample collection. LDO participated in microarray results interpretation, experimental design, and sample processing. DAW performed animal surgeries, exercised mice, took animal readings, and collected animal samples. JL performed histology, real-time RT-PCR, and cell area counts. JFM assisted with histology, data interpretation, and manuscript preparation. LJM wrote and utilized computer scripts for analysis of previously published array data. TQP participated in animal experiments and took animal readings. NMK participated in the interpretation of experimental results and in editing of the manuscript. HRG participated in the design and coordination of the study and helped to draft the manuscript. All authors read and approved the final manuscript.

## Supplementary Material

Additional file 1 **Supplementary Table 1 (Gene expression alterations in mice after ISO-treatment or exercise via swimming)**. The file provided is an Excel format that includes gene IDs, gene names and symbols, biological functions, fold-changes, statistical values. Click here for file
